# Curcumin protects against doxorubicin induced oxidative stress by regulating the Keap1-Nrf2-ARE and autophagy signaling pathways

**DOI:** 10.1007/s00213-023-06357-z

**Published:** 2023-03-22

**Authors:** Dehua Liao, Danggang Shangguan, Yi Wu, Yun Chen, Ni Liu, Jingyi Tang, Dunwu Yao, Yingrui Shi

**Affiliations:** 1 Department of Pharmacy, The Affiliated Cancer Hospital of Xiangya School of Medicine, Hunan Cancer Hospital, Central South University, Changsha, 410011 China; 2 Department of Radiation Oncology, The Affiliated Cancer Hospital of Xiangya School of Medicine, Hunan Cancer Hospital, Central South University, Changsha, 410011 China

**Keywords:** CUR, DOX, Autophagy, Keap1-Nrf2-ARE, Oxidative stress

## Abstract

**Background:**

Doxorubicin (DOX)-induced neurotoxicity is widely reported in previous studies. Oxidative stress has been validated as a critical event involved in DOX-induced neurotoxicity. As a selective autophagy adaptor protein, p62 is reported to regulate Keap1-Nrf2-ARE antioxidant pathway in response to oxidative stress. Curcumin (CUR) relieves depressive-like state through the mitigation of oxidative stress and the activation of Nrf2-ARE signaling pathway. However, the exact mechanism of CUR in alleviating DOX-induced neurotoxicity is still unknown.

**Materials and methods:**

The rats were randomly divided into three groups: control group, DOX group, and DOX + CUR group. At the end of 3 weeks, the behavior tests as sucrose preference test (SPT), forced swimming test (FST), and novelty-suppressed feeding test (NSFT) were performed to assess anxiety- and depression-like behaviors. The rats were sacrificed after behavior tests, and the brain tissues were collected for biochemical analysis.

**Results:**

It was observed that the administration of CUR could effectively reverse DOX-induced depressive-like behaviors. The exposure of DOX activated autophagy and increased oxidative stress levels, and the administration of CUR could significantly inhibit DOX-induced autophagy and suppress oxidative stress. More importantly, we also found that Keap1-Nrf2-ARE signaling pathway was involved in DOX-induced neurotoxicity and oxidative stress regulated by autophagy.

**Conclusion:**

Our study demonstrated that CUR could effectively reverse DOX-induced neurotoxicity through suppressing autophagy and mitigating oxidative stress and endoplasmic reticulum (ER) stress.

## Introduction

As an anthracycline antineoplastic agents, doxorubicin (DOX) is one of the most potent FDA-approved agents for the treatment of diverse types of tumors, such as ovarian, breast, gastrointestinal, Wilms’ tumor, Hodgkin’s and non-Hodgkin’s lymphoma, and pediatric leukemia (Bayles et al. 2022; Kuzu et al. 2018). The unexpected cytotoxicity like heart arrhythmias, neutropenia, and cardiotoxicity as well as neuron damage in brain was frequently observed, which hindered its clinical use (Bayles et al. 2022). DOX-induced neurotoxicity attracted more and more attention, which always accompanied with depression, anxiety, and impaired cognition function (Christie et al. 2012; Merzoug et al. 2011). Previous study has showed that decline in cognitive function was observed in more than 60% of breast cancer patients treated with DOX (Wefel et al. 2010). Thus, it is necessary for us to understand the molecular mechanism underlying DOX-induced neurotoxicity and to seek potential therapeutic strategies.

Numerous studies showed that the production of hydroxyl radicals, superoxide radicals, and hydrogen peroxide by DOX will always lead to oxidative stress (Abd-Ellatif et al. 2022; Mani et al. 2022). The accumulation of ROS will always disrupt the balance between oxidative and antioxidative, leading to oxidative stress. Previous studies have also widely reported that oxidative stress and reactive oxygen species (ROS) play a pivotal role in DOX-induced toxicity, including neurotoxicity (Cheung et al. 2013; Mizutani et al. 2005; Pal et al. 2012).

Autophagy, known as “self-eating”, degrades oxidative stress products and has positive effects on reducing oxidative damage (Filomeni et al. 2015; Huber et al. 2012). Oxidative stress, endoplasmic reticulum (ER) stress, or nutrient deprivation can activate autophagy, and the induction of autophagy acts to defend against oxidative stress and maintain cellular homeostasis (Eskelinen and Saftig 2009). As a selective autophagy adaptor protein, p62 can identify proteins that need to be degraded, and p62 is known to act in multiple critical signaling pathways. Numerous studies have showed that p62 can activate the nuclear factor (erytheroid-derived-2)-like 2 (Nrf2)-regulated antioxidant pathway by inhibiting kelch-like ECH associated protein 1 (Keap1) through a positive feedback mechanism (Jain et al. 2010; Lau et al. 2010). As a critical regulator of antioxidant response, Nrf2 plays a vital role in protecting neural cells from oxidative injury in the antioxidant system. Under normal physiological conditions, Nrf2 interacts with Keap1 to retain the quiescent state in the cytoplasm. Upon exposure to oxidative stress, Nrf2 dissociates from Keap1, transports into the nucleus, where it binds to phase 2 of the antioxidant response element (ARE), and leads to an increase in the expression of downstream protective proteins, such as heme oxygenase-1 (HO-1), NAD(P)H quinone oxidoreductase (NQO1), and glutamylcysteine ligase, modifier sub-unit (GCLM). Since oxidative stress is a major cause of neurotoxicity induced by DOX exposure, we hypothesized that the Keap1-Nrf2-ARE signaling pathway may be involved in the mechanism of DOX-induced neurotoxicity.

Curcumin (CUR), a yellow coloring agent extracted from curcuma longa, has pharmacological effects including antioxidant, anti-inflammatory, immunomodulatory, and neuroprotective activities (Aggarwal and Harikumar 2009; Maheshwari et al. 2006). Notably, CUR’s antioxidative properties hold a great deal of potential for neuroprotective effect. Previous studies have reported that the main mechanism of CUR in the treatment of oxidant stress-related diseases was the activation of Nrf2 (Madiha and Haider 2019; Yang et al. 2009). Our previous study have revealed that CUR relieves depressive-like state through the mitigation of oxidative stress and the activation of Nrf2-ARE signaling pathway (Liao et al. 2020b). By activating Nrf2-ARE signaling, CUR exerts its chemopreventive effects via the induction of antioxidant enzymes (Scapagnini et al. 2011). Soetikno et al. have also reported that CUR alleviates oxidative stress, inflammation, and renal fibrosis in remnant kidney through the Nrf2-keap1 pathway (Soetikno et al. 2013). Scapagnini et al. have reported that CUR strongly induces HO-1 expression and activity in different brain cells via the activation of Keap1-Nrf2-ARE signaling pathway (Scapagnini et al. 2011). Balogun et al. have also reported that CUR exhibited its chemopreventive effect by selectively activating the Nrf2-Keap1-ARE signaling pathway (Balogun et al. 2003).

Therefore, our present study aimed to explore the potential protective effects of CUR against DOX-induced neurotoxicity and depression-like behaviors in rats. In addition, to further investigate the possible molecular mechanisms underlying the therapeutic effects of CUR, we also explore whether the possible neuroprotective effect of CUR is associated with the inhibition of autophagy and Keap1-Nrf2-ARE signaling pathway.

## Materials and methods

### Animals

Male Sprague–Dawley rats (200–220 g, Hunan Cancer Hospital Animal Centre) were housed in standard conditions (23 ± 2 °C, 12 h light/dark cycle). Food and water were freely available in the whole experiment, except prior to sucrose preference test (SPT). This study was approved by the Animal Care and Use Committee of Hunan Cancer Hospital (protocol number 2020–059). All animal use procedures were carried out in accordance with the Regulations of Experimental Animal Administration issued by the State Committee of Science and Technology of the People’s Republic of China.

### Experimental design

Rats were randomly divided into three groups (*n* = 8): (1) control, (2) DOX, and (3) DOX + CUR. The dose and treatment duration were chosen based on our previous studies (Liao et al. 2018, 2020b). Normal saline was injected to rats in the control group (1.5 ml). For DOX group, the rats were injected with DOX every 2 days at a dose of 2.5 mg/kg, and a total of 7 injections were given to each rat. The DOX + CUR group received CUR (30 mg/kg) daily by gavage for 3 weeks starting 1 week before giving DOX. The body weight of each rat was monitored throughout the experiment, and drug doses were adjusted accordingly.

At the end of 3 weeks, behavioral tests were performed. After behavior tests, all rats were anesthetized with sodium pentobarbital (50 mg/kg) via intraperitoneal injection, and the brain tissues were rapidly removed, and the hippocampus was quickly dissected on the ice surface. Hippocampus is an important region of the brain, which functioned as learning and memory, especially for the short-term memory. Memory in hippocampus is actually the pivotal connection form between nerve cells, which is sensitive for the change of nervous system. Hence, the hippocampus in the brain region was widely used for the investigation of neurotoxicity in the animal model.

### Behavioral test

#### Sucrose preference test (SPT)

The SPT was performed using the same method as in previous study (Liao et al. 2018; Maniam and Morris 2010). Prior to testing conditions, all rats were separately housed for 48 h for habituation of surroundings. After deprivation of water for 14 h, two pre-weighted bottles containing water and 1% sucrose solution were randomly placed to each rat. After 1 h, the bottles were weighed again, and the consumed weights of 1% sucrose solution and tap water were recorded. The percentage preference for sucrose was calculated as follows: sucrose preference (%) = sucrose consumption/(sucrose consumption + water consumption).

#### Forced swimming test (FST)

The FST was performed as described previously with minor modifications (Kumar and Mondal 2016; Rinwa and Kumar 2013). The rats in our present study were separated and forced to swim in an open cylindrical container (45 cm height, 25 cm diameter) containing 35 cm of water (24 ± 1 °C) for a 15-min pretest. The rats were then dried and placed in their home cage. One day later, the rats were exposed to the same experimental conditions outlined earlier for a 5-min FST. The duration of immobility was scored by an experienced observer blind to the experimental design.

#### Novelty-suppressed feeding test (NSFT)

The NSFT was adapted from previous study (Hamani et al. 2010). Before NSFT, the rats were food-deprived for 24 h in their home cages. A small amount of food was placed on a piece of white paper (10 × 10 cm) which was placed in an open field (75 × 75 × 40 cm). The rats were allowed to explore the open field for 8 min. The time it took for the rat to approach and take the first bite of the food was defined as the latency time and was recorded in our study. Immediately afterwards, the animals were transferred to their home cage, and the total food intake for the next 5 min was also weighed to avoid the influence of the animals’ appetite.

### Histopathological examination

For histological tissue analysis, the brain tissues were fixed in 4% paraformaldehyde in PBS (pH 7.2) at room temperature overnight and processed routinely for embedding in paraffin. The paraffin tissues were sliced into 5-µm sections. Paraffin sections were stained with hematoxylin and eosin (H&E) for light microscopic examination. The histology was assessed by a pathologist who was blinded to the treatment groups.

### TUNEL staining

In our present study, the terminal deoxynucleotidyl transferase-mediated deoxyuridine triphosphate nick-end labeling (TUNEL) detection kit (KeyGen Biotech, Nanjing, China) was used to assess apoptosis. According to the manufacturer’s instructions, TUNEL-positive tubular cell numbers were counted at random in 20 non-overlapping cortical fields under 200 × magnification.

### Immunofluorescence staining

The procedures of immunofluorescence were performed as our previous study (Liao et al. 2020a). The hippocampus sections were incubated with primary antibodies against 4-hydroxynonenal (4-HNE) (1:100 Abcam, ab48506, Cambridge, MA) at room temperature. After washing with PBS for three times, the sections were then incubated with a secondary antibody: Cy3-conjugated goat anti-rat (1:300; Wuhan Goodbio Technology Co., ab6953, Ltd.). 4,6-Diamidino-2-phenylindole (DAPI) was used to stain the nucleus. The sections were then mounted with a fluorescent mounting medium and imaged. Colocalization of brains with 4-HNE and DAPI was observed under a fluorescence microscope. The cells with DAPI labeling (blue) overlapping with 4-HNE (green) in the brains were counted by an investigator blinded to the experimental design.

### Real-time PCR analysis

According to the manufacturer’s instructions, total RNA from the hippocampus tissues was extracted by trizol reagent (Invitrogen, USA). The gene expression of NQO-1, HO-1, and GCLM was determined in our present study. The RNA concentration was determined for quantity by using the spectrophotometry (Jingke, Ningbo, China). Quantitative polymerase chain reaction (PCR) was performed on Bio-Rad Cx96 Detection System (Bio-Rad, Hercules, CA, USA) using SYBR green PCR kit (Applied Biosystems Inc., Woburn, MA, USA) and gene-specific primers. The sequences of gene-specific primers are shown in Table 1. A 5 ng cDNA sample was used with 40 cycles of amplification. Each cDNA was tested in triplicate. *β*-actin was used as an internal standard to normalize the signals.

**Table 1:** Primers used in real-time PCR analyses of mRNA expression

Gene	Sense primer (5′-3′)	Antisense primer (5′-3′)
HO-1	TGCTCGCATGAACACTCTGGAGAT	ATGGCATAAATTCCCACTGCCACG
NQO-1	GTGAGAAGAGCCCTGATTGT	CCTGTGATGTCGTTTCTGGA
GCLM	TCGCCTACAAAAAGCGTCAC	CGCCAGGGAGGTACTCAAAC
*β*-actin	CATCCTGCGTCTGGACCTGG	TAATGTCACGCACGATTTCC

### Western blot analysis

For western blot analysis, total protein was isolated from the hippocampus, and the Bradford method was used to determined its concentration. The concentration of SDS-PAGE was 12%. After blocking, the blots were incubated with the respective primary antibody including GRP78 (Proteintech, 66,574–1-Ig, 1:1000), CHOP (Cell Signaling, 66,741–1-Ig, 1:1000), Nrf2 (Abcam, 16,396–1-AP, 1:200), Keap1 (Sigma-Aldrich, 60,027–1-Ig, 1:3000), LC3-I (Sigma-Aldrich, 18,725–1-AP, 1:1000), LC3-II (Sigma-Aldrich, 18,725–1-AP, 1:1000), Atg-5 (ABclonal Technology, 66,744–1-Ig, 1:1000), Atg-7 (ABclonal Technology, 10,088–2-AP, 1:1000), Becn1 (ABclonal Technology, 11,306–1-AP, 1:1000), p62 (Cell Signaling Technology, 66,184–1-Ig, 1:1000), and *β*-actin (Proteintech, 66,009–1-Ig, 1:4000). Following several washes, the membranes were then incubated with the second antibody (HRP-conjugated goat anti-mouse IgG) (AWS0002, 1:5000 dilution), and the signal was detected with an ECL kit (GE Healthcare Biosciences, Piscataway, NJ, USA) and was quantified using the Image J software.

### Statistical analysis

All results from the experiment were expressed as means ± standard deviation (SD) and analyzed using the SPSS version 13.0 software. All data were analyzed by one-way analysis of variance (ANOVA) with least significant difference (LSD) post-hoc multiple comparisons. The prior level of significance was established at *p* < 0 05.

## Results

### Effects of CUR pretreatment on DOX-induced body weight gain and behaviors

**Fig. 1:**
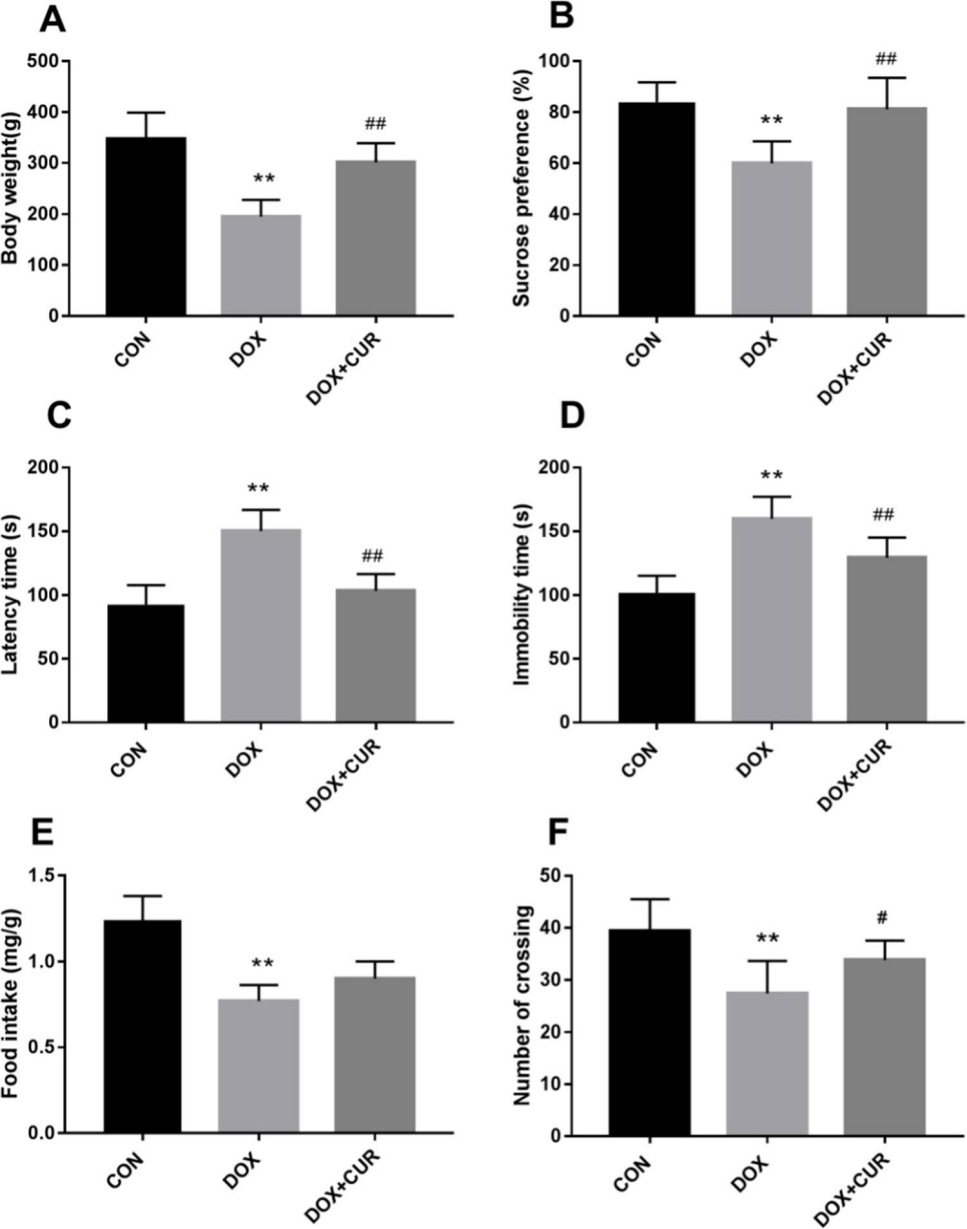
Body weight gain and behavior changes. Effects of DOX and dl-NBP on body weight gain (**A**), Sucrose preference in SPT (**B**), latency time in NSFT (**C**), immobility time in FST (**D**), food intake (**E**), and number of crossing (**F**) in OFT. Data are expressed as means ± SD (*n* = 7). *p < 0.05 and **p < 0.01 compared to the control group. ^#^*p* < 0.05 and ^##^*p* < 0.01 compared to the DOX group

As shown in Fig. 1A, the body weight of rats in DOX group shows a significant decrease when compared with the control group (*p* < 0.01), while the pretreatment of CUR had a positive effect on the body weight gain in DOX-treated rats (*p* < 0.01). In comparison with the rats in control group, DOX-treated rats exhibited decreased sucrose preference in SPT (Fig. 1B, p < 0.01), increased latency time in NSFT(Fig. 1C, p < 0.01), prolonged immobility time in the forced swimming test (FST) (Fig. 1D, p < 0.01), and decreased food intake (Fig. 1E, p < 0.01) and number of crossing (Fig. 1F, p < 0.01) in the open-field test (OFT). Fortunately, CUR could improve DOX-induced symptoms of depression, including increased sucrose preference in SPT (Fig. 1B, p < 0.01), decreased latency time in NSFT(Fig. 1C, p < 0.01) and immobility time in FST (Fig. 1D, p < 0.01), and number of crossing (Fig. 1F, p < 0.05) in OFT.

### Effects of DOX and CUR on histopathological changes and neural apoptotic markers

**Fig. 2:**
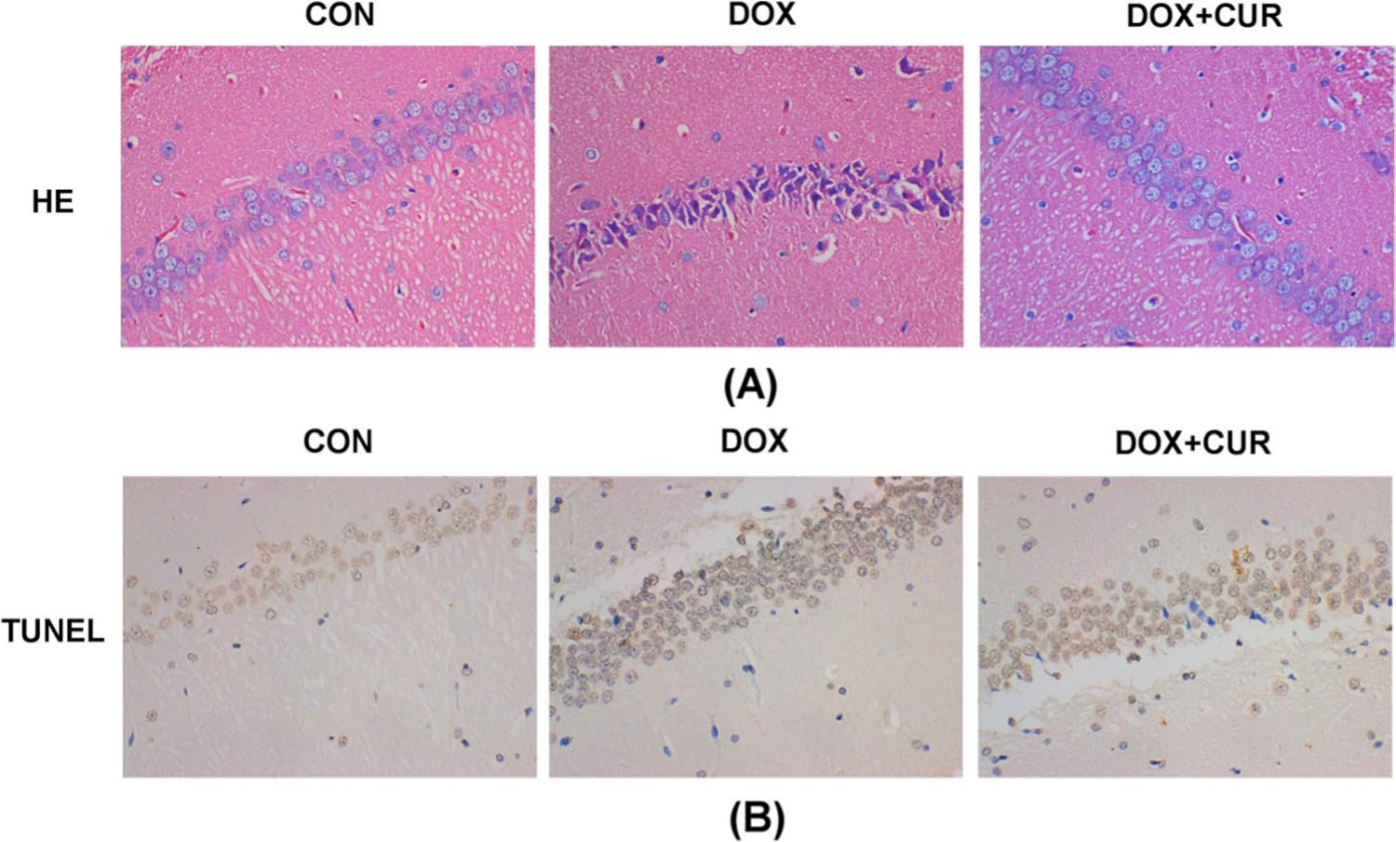
Effects of DOX and CUR on histopathological changes and apoptotic markers. HE staining of different group (**A**) and TUNEL staining of different group (**B**)

Histopathological alterations in hippocampus sections from different treated groups are shown in Fig. 2A. Compared with the control group, more frequent nuclear pyknosis of hippocampus was observed in DOX-treated group, while the pretreatment with CUR could ameliorate this histopathological alternation evoked by DOX. In our present study, TUNEL test was used to assess the severity of apoptosis in the hippocampus. As revealed in Fig. 2B, more TUNEL-positive cells are observed in the hippocampus of DOX group when compared with the control group. However, TUNEL-positive cells were significantly decreased when pretreated with CUR.

### Effects of DOX and CUR on oxidative stress

**Fig. 3:**
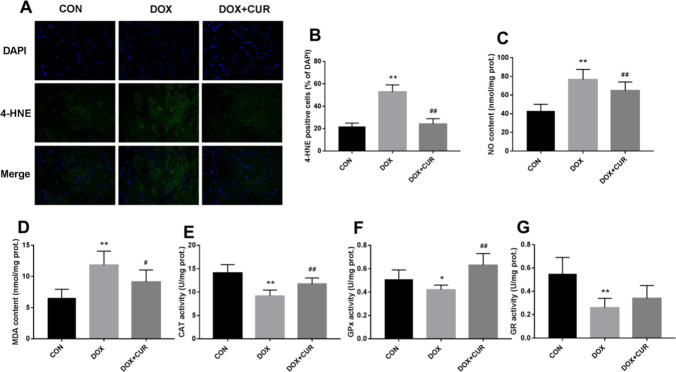
Effects of CUR on DOX-induced oxidative stress markers in the hippocampus. Immunofluorescence staining of 4-HNE (**A**), 4-HNE-positive cells (**B**), NO contents (**C**), MDA contents (**D**), CAT activity (**E**), GPx activity (**F**), and GR activity (**G**). Data are expressed as means ± SD (*n* = 7). *p < 0.05 and **p < 0.01 compared to the control group. ^#^*p* < 0.05 and ^##^*p* < 0.01 compared to the DOX group

The result of immunofluorescence staining is shown in Fig. 3A, and numerous 4-HNE-positive cells in hippocampus are observed in DOX-treated rats when compared with control group, and 4-HNE-positive cells are fewer in DOX + CUR group. For the quantitative result, we found that 4-HNE-positive cells were significantly increased in DOX treated rats (Fig. 3B, p < 0.01), while the pretreatment of CUR could significantly reverse this increase (Fig. 3B, p < 0.01). The parameters for oxidative/antioxidative system were assessed in our present study. The content of NO (Fig. 3C, p < 0.01) and MDA (Fig. 3D, p < 0.01) was significantly elevated in DOX group, and the pretreatment of CUR could significantly block this increasing. The activity of antioxidative parameter CAT (Fig. 3E, p < 0.01) and GPx (Fig. 3F, p < 0.05) was all significantly decreased in DOX, whereas the pretreatment of CUR induced a significant increase in the activity of CAT (Fig. 3E, p < 0.01) and GPx (Fig. 3F, p < 0.01). Although DOX induced a significant decrease in the activity of GR (Fig. 3G, p < 0.01), the activity of GR was not significantly increased in DOX + CUR group.

### Effects of DOX and CUR on ER stress

**Fig. 4:**
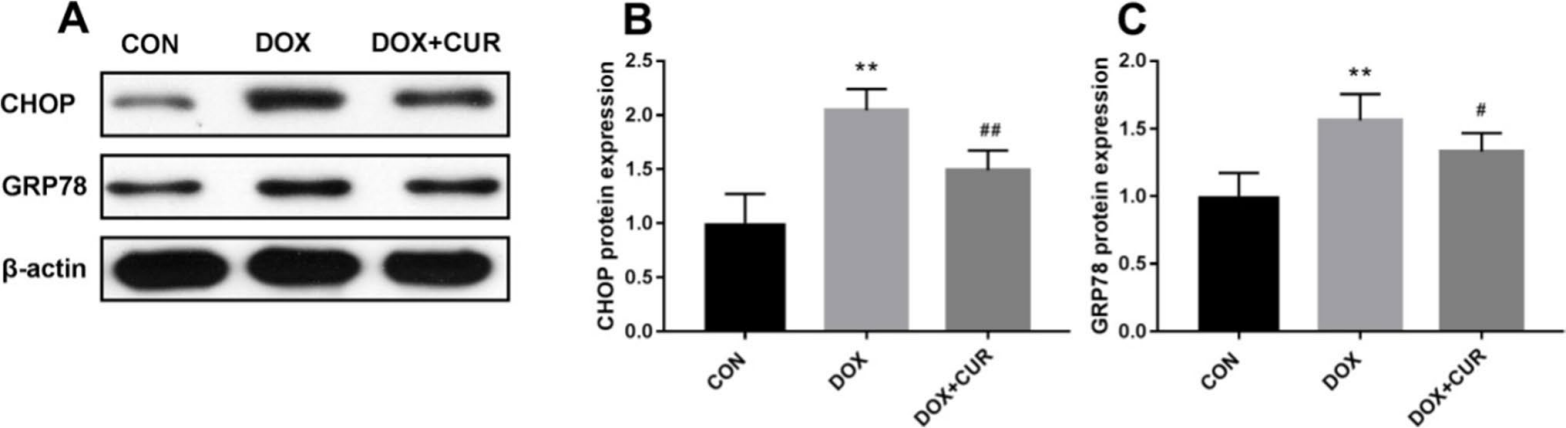
Effects of CUR on DOX-induced ER stress markers in the hippocampus. Protein expression of CHOP (**B**) and GRP78 (**C**). Data are expressed as means ± SD (*n* = 7). *p < 0.05 and **p < 0.01 compared to the control group. ^#^*p* < 0.05 and ^##^*p* < 0.01 compared to the DOX group

To explore the alternation of ER stress, the protein expression of GRP78 and CHOP was evaluated in our present study. In comparison with the control group, we found that the protein levels of CHOP (Fig. 4B, p < 0.01) and GRP78 (Fig. 4C, p < 0.01) were significantly increased after administration of DOX. Meanwhile, the upregulation of CHOP (Fig. 4B, p < 0.01) and GRP78 (Fig. 4C, p < 0.05) was effectively blocked by dl-NBP treatment.

### Autophagy-regulated proteins were increased in DOX-treated rats

**Fig. 5:**
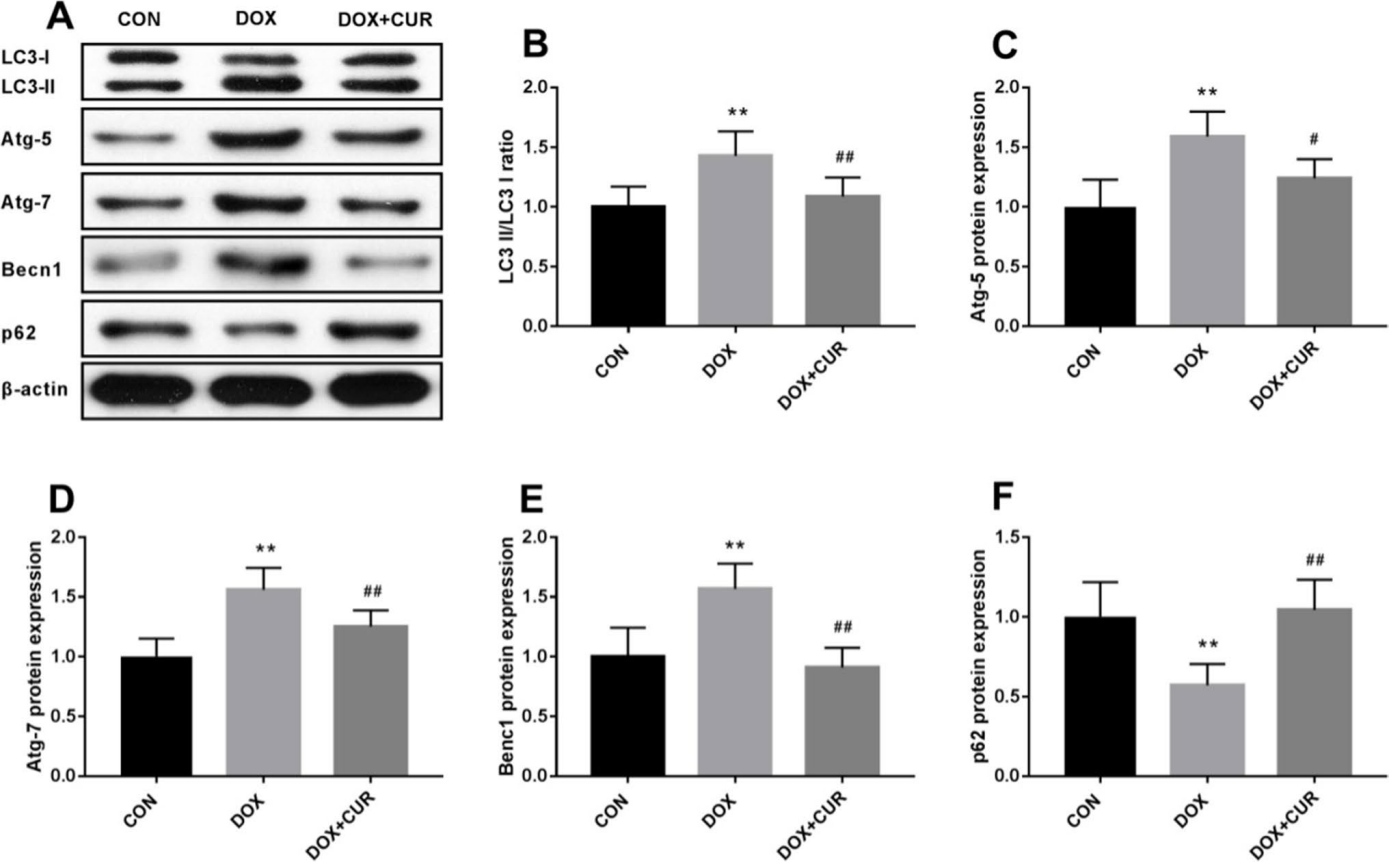
Effects of CUR on DOX-induced autophagy in the hippocampus. LC3-II/LC3-I ratio (**B**), protein expression of Atg-5 (**C**), protein expression of Atg-7 (**D**), protein expression of Benc1 (**E**), protein expression of p62 (**F**)

In our present study, the effect of DOX on the activity of autophagy was assessed. As autophagy-related proteins, LC3II, Atg-5, Atg-7, and Becn1 were detected to assess autophagic activity in the hippocampus of rats exposed to DOX. As shown in Fig. 5B, the ratio of LC3-II/LC3-I in hippocampus is significantly increased after exposure to DOX (*p* < 0.01), and the administration of CUR could effectively reversed this elevation (*p* < 0.01). The protein expression levels of Atg-5 (Fig. 5C, p < 0.01), Atg-7 (Fig. 5D, p < 0.01), and Becn1 (Fig. 5E, p < 0.01)were upregulated after exposure to DOX, and the administration of CUR effectively moderated this phenomenon. p62, which is another type of autophagy-adaptor protein, has been documented to associate with Nrf2 signaling and autophagy via binding with Keap1. Hence, the protein expression of p62 was also evaluated in our present study. In comparison with the rats in control group, DOX induced a significant decrease of p62 (Fig. 5F, p < 0.01), while the pretreatment of CUR could significantly reverse this phenomenon (Fig. 5F, p < 0.01). Taken together, the above-mentioned results indicated that the occurrence of autophagy during DOX-induced neurotoxicity and the administration of CUR could alleviate autophagy.

### Effects of CUR on the activation of p62-KEAP1-Nrf2 in DOX-treated rats

**Fig. 6:**
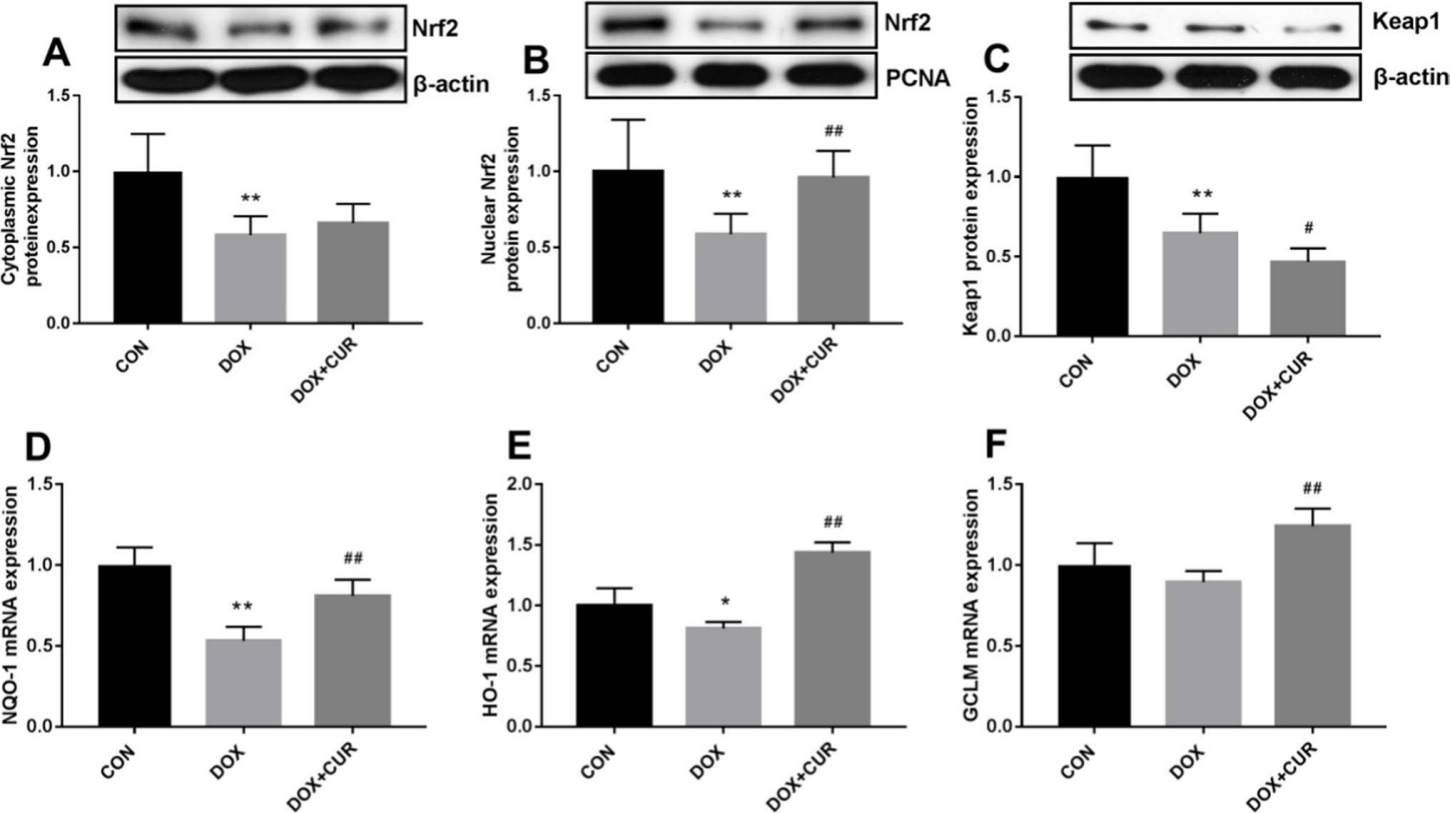
Effects of CUR on the activation of Keap1-Nrf2 in DOX-treated rats. Protein expression of Nrf2 in cytoplasmic (**A**), protein expression of Nrf2 in nuclear (**B**), protein expression of Keap1 (**C**), mRNA expression of NOQ-1in the hippocampus (**D**), mRNA expression of HO-1in the hippocampus (**E**), mRNA expression of GCLM in the hippocampus (**F**)

In our present study, the protein expression of Nrf2 and Keap1 after DOX exposure with or without CUR pretreatment was determined by western blotting. In comparison with the control group, the protein level of Nrf2 in cytoplasmic (Fig. 6A, p < 0.01) and nuclear (Fig. 6B, p < 0.01) was all significantly decreased for DOX group. We also found that Nrf2 in nuclear was significantly increased in the CUR pretreated rats compared to the DOX group (Fig. 6B, p < 0.01), but not Nrf2 in cytoplasmic. For the rats exposed to DOX, the expression of Keap1 was significantly decreased (Fig. 6C, p < 0.01), and we also observed that CUR induced an even more significant decrease (Fig. 6C, p < 0.05). In addition, for the downstream molecules of Nrf2 signaling pathway, the mRNA levels of NQO-1, HO-1, and GCLM were also evaluated in our present study. The mRNA expression of NQO-1 (Fig. 6D, p < 0.01) and HO-1 (Fig. 6E, p < 0.05) was all significantly decreased in DOX group, and the pretreatment of CUR could significantly reverse these decrease (Fig. 6E, p < 0.01). Although the mRNA level of GCLM was not significantly decreased after exposure to DOX, the administration of CUR induced a significant elevation (Fig. 6F, p < 0.01).

## Discussion

Our present study demonstrated that the administration of CUR exhibited neuroprotective effect in DOX-induced neurotoxicity model. The behavior test (SPT, FST, NSFT, OPT) results showed that pretreatment with CUR normalized behavioral changes in rats exposed to DOX. We found that CUR could significantly attenuate oxidative stress and ER stress in the hippocampus of rats when exposed to DOX. More importantly, we also found that the potential neuroprotective mechanism of CUR might be related to the suppression of autophagy and the activation of KEAP1-Nrf2-ARE signaling pathway.

As it is known to us, DOX is an essential agent for the treatment of numerous malignant tumors. The long-term use of DOX will always induce dose-related neurotoxicity, and depression and anxiety are the specific performance of neuropsychiatric diseases (Rocha et al. 2018). The behavior tests were performed to access the depression and anxiety status in our present study. For rats exposed to DOX, decrease sucrose preference in SPT, prolong immobility time in FST, and decrease number of crossing and food intake in OFT were observed in our present study. However, pretreatment with CUR could effectively normalized behavioral changes for rats which exposed to DOX. Numerous studies have reported that CUR exhibited neuroprotective action in animal model of neurotoxicity. Madiha et al. (Madiha and Haider 2019) have reported that pre- and post-treatment with CUR produced antidepressant like effects in FST by decreasing immobility time, increasing swimming time and number of jumps. Kumar et al. (2009) have also found that chronic administration of CUR was able to reverse the behavior of cognitive deficit, emphasizing its potential neuroprotective efficiency against aluminum-induced neurotoxicity. According to the behavioral investigations, Pyun et al. (2014) have also reported that CUR displayed protective effects against alcohol-induced neuronal damage. CUR could also effectively improve ambulation number and ambulation distance in nicotine-induced neurotoxicity model (Motaghinejad et al. 2017). Our previous study also found that chronic administration of CUR could effectively attenuate chronic unpredictable mild stress-induced depressive-like behaviors (Liao et al. 2020b).

An over production of ROS plays vital role in the observed toxicity of brain tissue. DOX increases oxidative stress and reduces the total antioxidant capacity, triggering neurodegeneration and neuronal cell death that result in neurobehavioral alterations (Aziriova et al. 2014; El-Agamy et al. 2018). Mahmoodazdeh et al. (2020) have reported the alteration in the levels of various aspects of oxidative stress including elevated levels of ROS, NO, and MDA in the DOX-treated dorsal root ganglia neurons. Kuzu et al. (2018) have reported that oxidative stress, inflammation, and apoptosis were elevated for the rats exposure to the DOX. In line with these findings, our present study revealed that the levels of oxidative stress parameters (MDA, NO, CAT, GPx, GR) were significantly increased and the antioxidant enzymes were significantly decreased in the hippocampus of DOX-treated rats. Fortunately, in this work, we clearly demonstrated the capability of CUR in attenuating DOX-induced oxidative stress and exhibiting antioxidant effect. This is consistent with previous studies. Benzer et al. (2018) have reported that CUR ameliorates DOX-induced cardiotoxicity by abrogation of oxidative DNA damage and protein oxidation in rats. Namdari and Eatemadi (2017) have reported that the protective effect of CUR on acute DOX-induced toxicity has been related to its antioxidant capacity against lipid peroxidation.

ER stress signaling is usually protective against accumulation of misfolded proteins, thereby enabling cells to survive various type of stress. Previous study has reported that CHOP and GRP78 pathways could be activated by ER stress and thereby mediate apoptosis (Tabas and Ron 2011). In addition, accumulating studies demonstrated that DOX-induced ER stress subsequently cause myocardial cell death through this apoptosis pathway (Wang et al. 2012). Hence, the protein levels of ER stress markers (CHOP and GRP78) were evaluated in our present study. We found that the administration of DOX induced significant neurotoxicity with increased protein expression of CHOP and GRP78, and the pretreatment of CUR could effectively reverse these upregulations. In line with our findings, Zhou et al. (2018) have reported that protein levels of ER stress markers (CHOP and GRP78) were significantly increased in DOX-treated rats. Yarmohammadi et al. (2021) have reviewed that endoplasmic reticulum stress in doxorubicin-induced cardiotoxicity may be therapeutically targeted by natural and chemical compounds. Li et al. (2015) have reported that curcumin attenuated glutamate neurotoxicity by inhibiting ER stress-associated TXNIP/NLRP3 inflammasome activation via the regulation of AMPK. Moreover, targeting ER stress has been considered as a potential strategy of CUR in the management of neurodegenerative disorders (Shakeri et al. 2019).

Autophagy is a biological process of self-repair that is involved in cellular growth, metabolism, and defences against oxidative stress. In addition, the activation of autophagy can degrade proteins against ER stress-induced toxicity (Eskelinen and Saftig 2009). To confirm the effect of DOX on the activity of autophagy, the ratio of LC3-II/LC3-I was detected to measure autophagic activity in hippocampus tissue, as well as the autophagy-related proteins p62, Atg5, Atg7, and Becn1. As shown in Fig. 3, the ratio of LC3-II/LC3-I is significantly increased after exposure to DOX. In addition, we also found that the expression levels of p62, Atg5, Atg7, and Becn1 increased gradually after injected with DOX. These results indicated the occurrence of autophagy during DOX-induced neurotoxicity. It is interesting that we also found that CUR could effectively decrease these autophagy-related proteins after exposure to DOX. Katamura et al. (2014) have reported that CUR attenuates DOX-induced cardiotoxicity by inducing autophagy via the regulation of JNK phosphorylation. Yu et al. (Wang et al. 2012) have reported that CUR rescued against DOX-induced cardiac injury probably through regulation of autophagy and pyroptosis in a mTOR-dependent manner.

The activation of Nrf2-ARE pathway is the main mechanism of cell defense against oxidative stress and ER stress, which enhances the antioxidant capacity of cells. Keap1-Nrf2 antioxidant defense pathway has been well-known to afford neuroprotection. Furthermore, autophagy signaling pathway is also involved in the oxidative stress response. Previous studies have reported that p62 provided a key link between autophagy and the Keap1-Nrf2 signaling pathway under oxidative stress. p62 could interact with Keap1 and disrupt the association between Keap1 and Nrf2, causing the stabilization and nuclear accumulation of Nrf2. Shen et al. (2018) have reported that p62-keap1-Nrf2 antioxidant pathway was primarily activated in the early stage of APAP hepatotoxicity. Tan et al. (2020) have reported that luteolin exerts neuroprotection via modulation of the p62-Keap1-Nrf2 pathway in intracerebral hemorrhage. In addition, numerous studies have also showed that the activation of autophagy and the p62-Keap1-Nrf2-positive feedback loop are protective mechanisms that ameliorate the development of neurodegenerative diseases (Buratta et al. 2020; Lattante et al. 2015). In our present study, we focused on the p62-Keap1-Nrf2 signaling pathway to investigate the mechanisms of oxidative injury and the antioxidant system. We found that autophagy was increased in DOX-treated rats, p62 was upregulated in the hippocampus under DOX-induced oxidative stress conditions, and pretreatment with CUR could significantly decrease this autophagy. As a substrate for autophagy, p62 maybe also involved in the regulation of autophagy through the Keap1-Nrf2-ARE signaling pathway, where it plays a pivotal role in preventing oxidative damage and alleviating ER stress. The protein expression of Nrf2 and Keap1 was determined in our study in an attempt to elucidating the mechanism by which CUR reversed DOX-induced antioxidant enzyme activities. The results showed that the protein level of Nrf2 in cytoplasmic and nuclear was all significantly decreased for DOX group, and Nrf2 in nuclear was significantly increased in the CUR pretreated rats compared to the DOX group, but not Nrf2 in cytoplasmic. The same trends were also observed for the protein expression of Keap1. The pretreatment of CUR could significantly reverse DOX-induced decrease of the mRNA expression of NQO-1and HO-1, which are downstream molecules of Nrf2 signaling pathway. Consistent with our results, Liao et al. (2019) have reported that p62/SQSTM1 protects against cisplatin-induced oxidative stress in kidneys by mediating the cross talk between autophagy and the Keap1-Nrf2 signaling pathway. He et al. (2012) have reported that CUR attenuated glucose intolerance by decreasing the oxidative stress and improving the nuclear level of NRF2 and its downstream target HO-1 in a high-fat diet mice model. He et al. (Han et al. 2012) have indicated that CUR has the potential for use as an autophagic-related antioxidant for prevention and treatment of oxidative stress. Tu et al. (Di Tu et al. 2020) have also reported that CUR could alleviate the development of membranous nephropathy by inducing autophagy and alleviating renal oxidative stress through the PI3K/AKT/mTOR and Nrf2/HO-1 pathways. In addition, oxidative stress, imbalance between autophagy and apoptosis systems, and Nrf2 activation are the main mechanisms of DOX-induced cardiotoxicity (Mohajeri and Sahebkar 2018).

## Conclusion

**Fig. 7:**
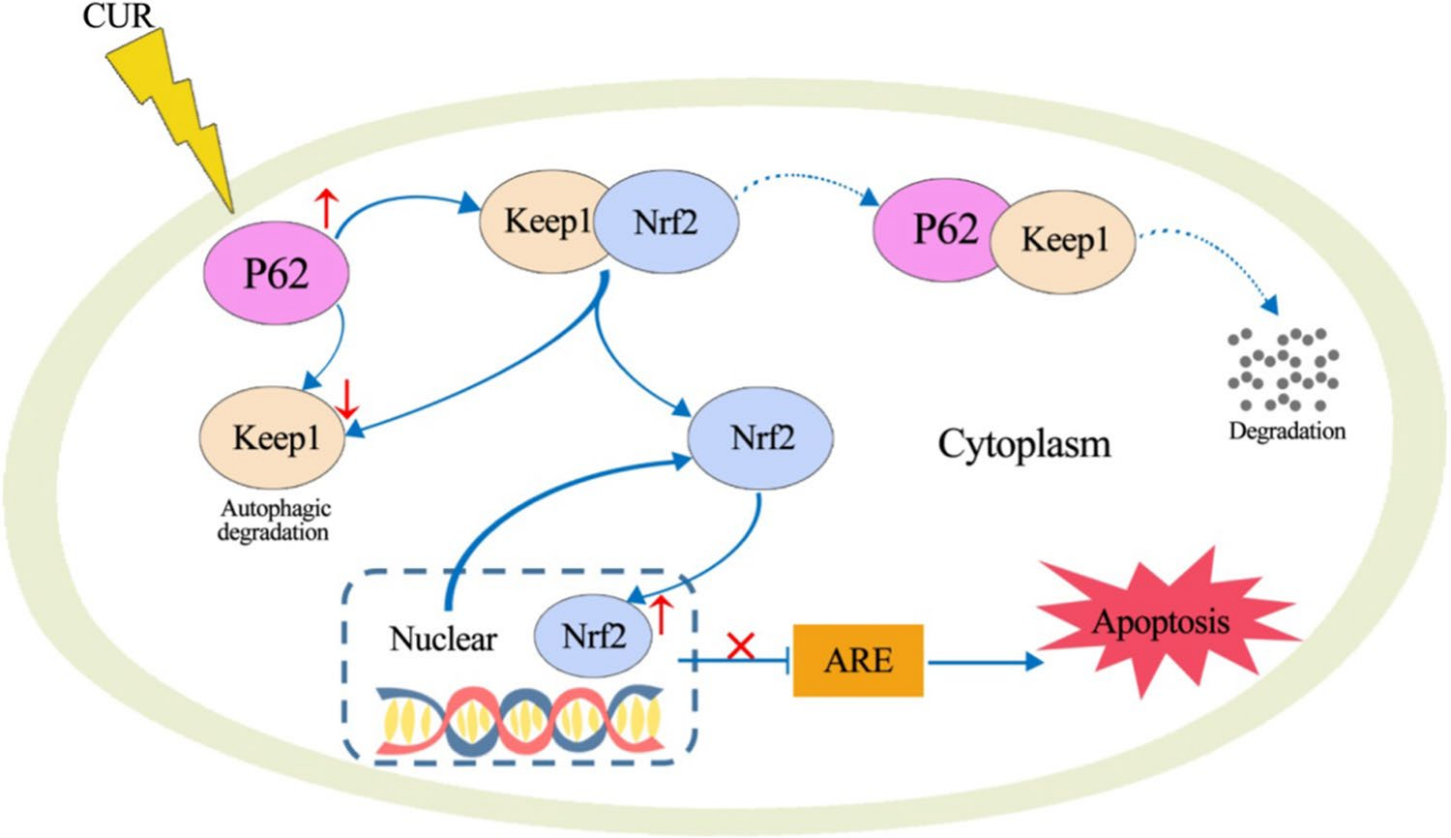
Proposed mechanism of CUR in protecting against DOX-induced neurotoxicity

Our present study has demonstrated that CUR exhibited great potential to reverse the depressive-like behavior in DOX-treated rats. DOX-induced excessive oxidative stress causes the upregulation of autophagy, and autophagy acts as an antioxidant feedback response activated by the p62-Keap1-Nrf2 feedback loop. The possible mechanism under behavior-modulating and neuroprotective effects of CUR is the activation of p62-Keap1-Nrf2 signaling pathway (Fig. 7).

## Data Availability

The data used to support the findings of this study are available from the corresponding author upon request.
